# Evolocumab enables rapid LDL-C reduction and inflammatory modulation during in-hospital stage of acute coronary syndrome: A pilot study on Chinese patients

**DOI:** 10.3389/fcvm.2022.939791

**Published:** 2022-08-09

**Authors:** Ziwei Ou, Zaixin Yu, Benhui Liang, Lin Zhao, Jianghua Li, Xinli Pang, Qiyun Liu, Cong Xu, Shaohong Dong, Xin Sun, Tangzhiming Li

**Affiliations:** ^1^Department of Cardiology, Xiangya Hospital, Central South University, Changsha, China; ^2^Department of Cardiology, Xiangya Third Hospital, Central South University, Changsha, China; ^3^Department of Cardiology, Shenzhen Cardiovascular Minimally Invasive Medical Engineering Technology Research and Development Center, Shenzhen People's Hospital (The Second Clinical Medical College, The First Affiliated Hospital, Southern University of Science and Technology, Jinan University), Shenzhen, China

**Keywords:** evolocumab, acute coronary syndrome, atherogenic lipids, inflammatory cytokines, Chinese patients

## Abstract

**Background and aims:**

Proprotein convertase subtilisin/kexin type 9 (PCSK9) has long been considered a key regulator in lipid metabolism. Its role as a potential player in immune response has recently earned much attention. However, the effects of evolocumab, an approved PCSK9 monoclonal antibody, on lipid reduction and inflammation regulation in Chinese patients with acute coronary syndrome (ACS) during their in-hospital stage after an index event are not well known.

**Methods:**

We conducted a case-crossover pilot study (http://www.clinicaltrials.gov/, NCT04730648) involving 31 patients hospitalized for ACS with elevated low-density lipoprotein cholesterol (LDL-C) level (≥70 mg/dL despite high-intensity statin) and 8 age- and gender-matched patients without coronary heart disease (CHD) as the baseline control. The patients with ACS received one dose of subcutaneous evolocumab (140 mg) on top of 10 mg/day rosuvastatin during hospitalization. Blood samples at baseline and 72 h post-evolocumab administration were collected for lipid and cytokine assessments.

**Results:**

The patients without CHD shared similar risk factors and LDL-C levels with the patients with ACS but exhibited a more activated inflammatory status. After single-dose in-hospital evolocumab, the median LDL-C level of patients with ACS decreased from 109.0 to 41.4 mg/dL as early as 72 h, accompanied with reductions in other atherogenic lipids. Systemic inflammatory pattern was also altered, rendering a decrease in pro-inflammatory and anti-inflammatory cytokines.

**Conclusion:**

In this case-crossover study of the effect of PCSK9 antibody among Chinese patients, evolocumab on top of high-intensity statin during hospitalization led to a remarkable and rapid reduction in atherogenic lipids and an alteration in inflammatory status at early-stage post-ACS.

## Introduction

Current lipid management guidelines recommended the administration of high-intensity statin to patients with acute coronary syndrome (ACS) for the prevention of recurrent ischemic events (1, 2). However, the residual cardiovascular risk remains high in statin-treated patients with ACS, particularly at the early stage of a post-index event (3), owing to the sustained systemic inflammatory state and unsatisfactory low-density lipoprotein-cholesterol (LDL-C) level (4). Given the poor adherence to high-intensity statin therapy and the suboptimal lipid-lowering and inflammation-controlling effects of statins within 30 days after an index event in patients with ACS (5–8), new agents should be considered for acute use to reduce LDL-C and alleviate systemic inflammation more rapidly and potently.

Proprotein convertase subtilisin/kexin type 9 (PCSK9) is a serine protease expressed in the liver and is known as a key regulator of low-density lipoprotein (LDL) metabolism. PCSK9 redundance can induce LDL-C accumulation in the circulation and lead to hyperlipidemia by competitively binding to LDL receptor (LDLR) on the cell surface and subsequently causing LDLR internalization and degradation (9). In addition to lipid metabolism, PCSK9 is also associated with many other physiological processes (10), among which immune response is of great attention (11, 12).

A growing body of evidence has shown that neutralizing monoclonal antibodies against PCSK9, such as alirocumab and evolocumab, can reduce the long-term incidence of major adverse cardiovascular events (MACEs) in patients with established atherosclerotic cardiovascular disease (13–16). Recent studies also focused on the efficacy of LDL-C reduction with evolocumab in patients with ACS, supporting the benefit of the early initiation of PCSK9 monoclonal antibody for patients with ACS (17, 18). However, the effect of PCSK9 monoclonal antibody on inflammatory pattern during acute-phase post-ACS remains unclear, and little is known about the efficacy of evolocumab in the Chinese population.

Against this background, we conducted this pilot trial to assess the lipid-lowering and inflammation-regulating effects of the in-hospital use of evolocumab on top of high-intensity statin in Chinese patients with current ACS.

## Methods

### Study design and patients

This study (Acute Myocardial Infarction and Unstable Angina with PCSK9 Inhibitor Usage Study, AMONG-US) employed a case-crossover design and included patients with recent ACS (<1 week) whose LDL-C levels were higher than guideline-recommended targets (2) despite high-intensity statin (10 mg rosuvastatin) therapy. The protocol was approved by the institutional ethics committee of Shenzhen People's Hospital, and all study subjects signed informed consent. The protocol and study design were prospectively described on ClinicalTrials.gov under the ID: NCT04730648. We complied with the guidelines of Consolidated Standards of Reporting Trials for clinical trials.

Screening was performed in hospitalized patients undergoing percutaneous coronary intervention (PCI) from April 2021 to December 2021 at the Department of Cardiology, Shenzhen People's Hospital. Thirty-one patients with recent ACS were enrolled in the study. ACS diagnosis was based on clinical symptoms (i.e., chest pain), cardiac biomarker values (i.e., cardiac troponin), changes in electrocardiography, and intracoronary thrombus identified by angiography (19). Eight patients complaining about chest pain at admission with comparable principal risk factors but classified as non-coronary heart disease (CHD) after coronary angiogram evaluation were recruited as controls for comparison.

The study drug (evolocumab, Repatha^®^, 140 mg, single dose) was administered subcutaneously to patients with ACS after PCI as early as possible. The patients received rosuvastatin (10 mg/day) throughout the study.

### Exclusion criteria

Patients with autoimmune diseases (e.g., Crohn's disease, systemic lupus), recent infectious diseases, estimated glomerular filtration rate <30 mL/min/1.73 m^2^, or under immunosuppressive drug therapy (e.g., oral steroids, non-steroidal anti-inflammatory agents, and cyclosporine) were excluded from this study.

### Blood sample collection and laboratory

Fasting blood samples were obtained at baseline (before evolocumab administration) from all subjects and at 72 h post-evolocumab administration from the patients with ACS for the assessment of fasting lipids and circulating inflammatory markers. For measurement of fasting lipids, blood samples were collected and centrifuged at 4,000 × g for 5 min, and serum aliquots were stored at −80°C for further analysis. Serum LDL-C, high-density lipoprotein-cholesterol (HDL-C) and small dense low-density lipoprotein cholesterol (sdLDL-C) levels were measured directly using homogenous assay, and serum triglyceride (TG) and total cholesterol (TC) levels were detected using a direct colorimetric assay by the central laboratory of Shenzhen People's Hospital. Levels of Apolipoprotein B (ApoB), apolipoprotein E (ApoE) and apolipoprotein A1 (ApoA1) were measured *via* turbidimetric inhibition immunoassay.

For evaluation of circulating biomarkers, EDTA-anticoagulated blood samples were centrifuged within 30 min of collection at 3,000 × g for 10 min, and plasma aliquots were stored at −80°C until further analysis. The level of PCSK9 and two platelet activation markers in plasma, soluble P-selectin and platelet factor-4 (PF-4), were measured in duplicate using commercially available enzyme-linked immunosorbent assay kits (Cloud-Clone) per the manufacturer's suggestion.

Bio-Plex Pro Human Cytokine 27-plex Immunoassay Kit (Bio-Rad), a human cytokine standard, was utilized to detect the plasma concentration of the following 27 cytokines based on the Luminex 200 system: interleukin-6 (IL-6), regulated upon activation normal T cell expressed and secreted factor (RANTES), vascular endothelial growth factor (VEGF), granulocyte-macrophage colony-stimulating factor (GM-CSF), interleukin-1 beta (IL-1β), interleukin-13 (IL-13), platelet-derived growth factor-BB (PDGF-BB), interleukin-12 p70 (IL-12p70), interleukin-1 receptor antagonist (IL-1ra), interleukin-10 (IL-10), interleukin-15 (IL-15), interferon gamma (IFN-γ), interleukin-5 (IL-5), monocyte chemoattractant protein-1 (MCP-1), interleukin-2 (IL-2), interleukin-7 (IL-7), interleukin-8 (IL-8), eotaxin, interleukin-4 (IL-4), tumor necrosis factor alpha (TNF-α), macrophage inflammatory protein-1 beta (MIP-1β), interleukin-9 (IL-9), fibroblast growth factor basic (basic FGF), interleukin-17 (IL-17), interferon-inducible protein-10 (IP-10), macrophage inflammatory protein-1alpha (MIP-1α), and granulocyte-colony stimulating factor (G-CSF).

### Statistical analysis

Quantitative variables are shown as mean ± standard deviation (SD) for normally distributed data or median with interquartile range for abnormally distributed data. Student's *t*-test or Mann–Whitney *U*-test was used for the comparison between two independent groups as appropriate. Wilcoxon matched-pairs signed rank test was used for the comparison between self-controlled paired groups. Categorical variables are shown as numbers and percentages, and Chi-square test was performed for comparisons. Statistical analysis was performed with GraphPad Prism 8, and figures were constructed with Adobe illustrator. *P* < 0.05 was considered statistically significant.

## Results

### Clinical characteristics of patients without CHD and patients with ACS

**Figure 1:**
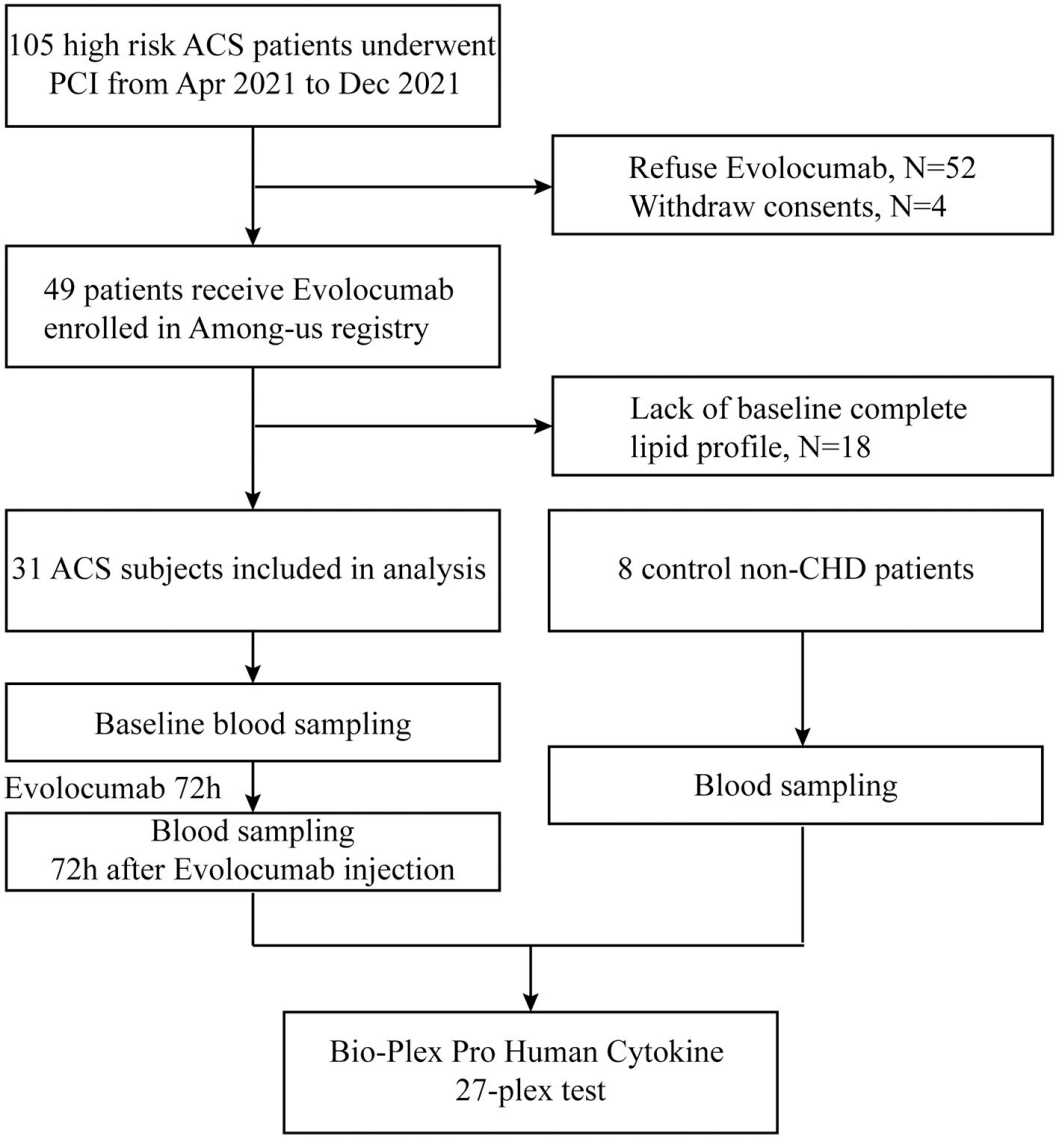
Study flowchart. Definition of acute coronary syndrome (ACS) is clarified in Methods. Baseline blood samples were collected after percutaneous coronary intervention or coronary angiogram. Non-CHD indicates patients without coronary heart disease.

Out of 105 patients screened, 49 patients with ACS were approached for enrollment and received evolocumab (1 dose, 140 mg) during hospitalization. Thirty-one patients were included in this study based on information integrity, and eight age-, gender-, and complication-matched patients who did not meet the diagnostic criteria of CHD by coronary angiogram evaluation were included for comparison (Figure 1).

Eligible patients with ACS did not differ from those without CHD in terms of age, gender, and body mass index. Overall, the mean age was 55.7 ± 9.6 years, 76.9% of patients were male, and the median body mass index was 25.4 (23.4, 27.5) kg/m^2^. The baseline clinical features were mostly well balanced between the two groups in the light of risk factors (frequencies of diabetes mellitus, hypertension, and smoking), renal function, platelet activation state (indicated by the level of soluble P-selectin and platelet factor-4), and lipid burden. The average plasma level of PCSK9 was higher in patients with ACS compared to patients without CHD (337.7 ± 83.3 vs. 237.5 ± 81.5 ng/mL, *p* = 0.0076) (Supplemental Figure 1). All patients were on high-intensity statin at admission, and the mean LDL-C level at baseline was 115.3 ± 35.3 mg/dL. The baseline characteristics of both groups are summarized in Table 1.

**Table 1:** Baseline characteristics in patients with non-CHD and ACS.

	**Non-CHD** **(*n* = 8)**	**ACS** **(*n* = 31)**	***P*** **-value**
Age (years)	58 [55, 60]	56 [48, 65]	0.6020
Male gender, *n* (%)	5 (63)	25 (81)	0.2775
Body mass index (kg/m^2^)	26.3 [23.3, 27.9]	25.0 [23.4, 27.5]	0.6889
Diabetes mellitus, *n* (%)	5 (63)	10 (32)	0.2202
Arterial hypertension, *n* (%)	4 (50)	19 (61)	0.5627
Active smoking, *n* (%)	4 (50)	15 (48)	0.1782
Statin treatment, *n* (%)	8 (100)	31 (100)	-
eGFR (ml/min/1.73 m^2^)	86.9 ± 12.3	84.1 ± 20.8	0.7267
Soluble P-selectin (ng/mL)	28.67 [25.28, 32.16]	26.03 [20.04, 33.06]	0.4297
Platelet factor-4 (ng/mL)	0.95 [0.69, 1.28]	1.38 [1.15, 1.65]	0.0128
TG (mg/dL)	139.1 [111.2, 194.9]	152.4 [113.4, 217.1]	0.6634
TC (mg/dL)	159.5 [104.7, 209.3]	194.1 [159.3, 221.9]	0.0773
HDL-C (mg/dL)	34.02 [26.39, 47.36]	36.73 [32.86, 46.39]	0.3965
LDL-C (mg/dL)	92.59 [59.63, 127.0]	109.0 [90.85, 144.6]	0.1212

*Data expressed as median [interquartile range], mean ± SD or number (percentage). Differences were tested using the Mann Whitney test, unpaired t-test or Chi-square test, as appropriate*.

*CHD, coronary heart disease; ACS, acute coronary syndrome; eGFR, estimated glomerular filtration rate; TG, triglyceride; TC, total cholesterol; HDL-C, high density lipoprotein cholesterol; LDL-C, low density lipoprotein cholesterol*.

### Inflammatory patterns in non-CHD and ACS patients

**Figure 2:**
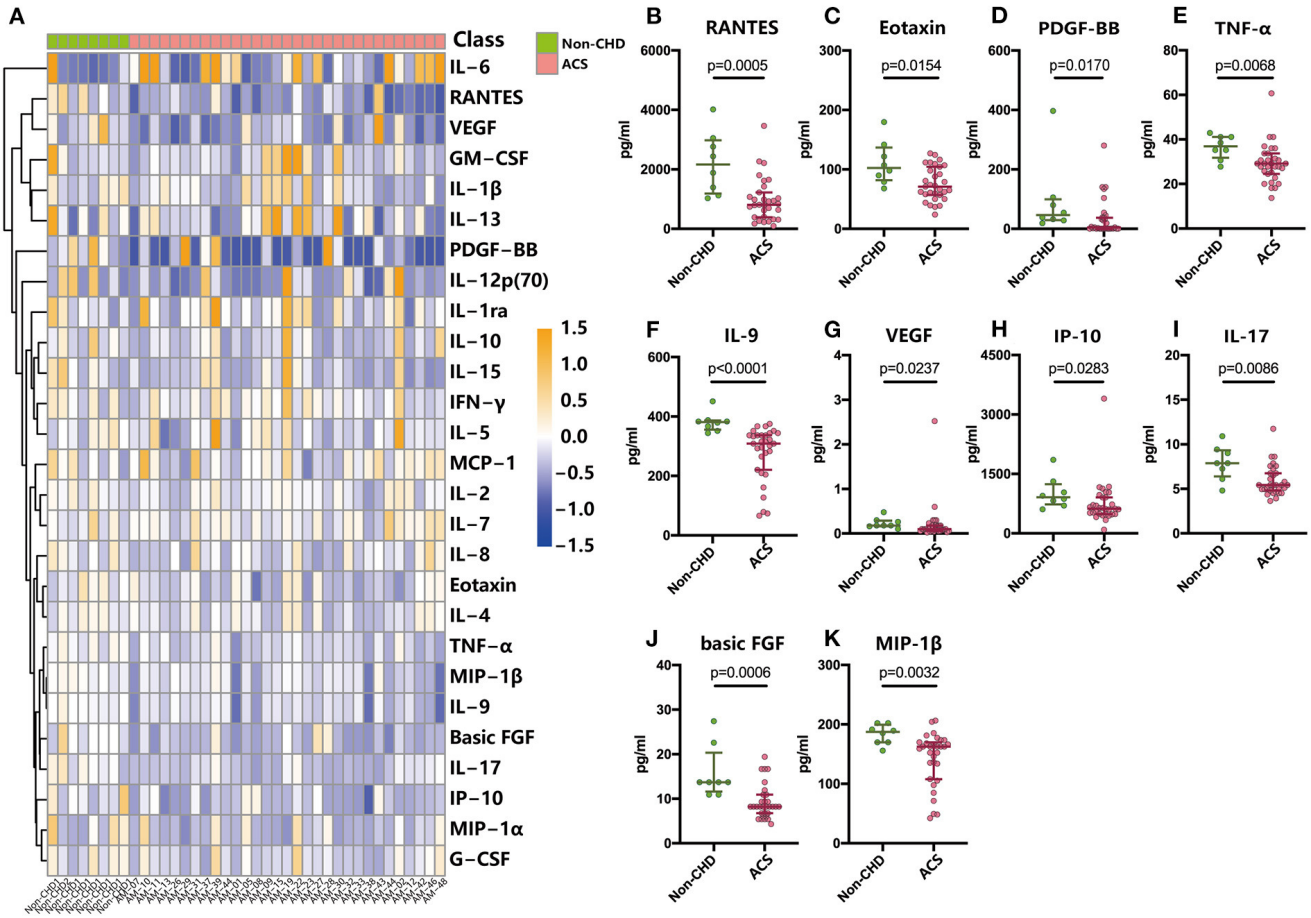
Baseline plasma cytokine and chemokine levels in patients with non-CHD and ACS. Cytokine and chemokine levels were measured using Luminex assay in 8 patients without CHD and 31 patients with ACS. **(A)** Heat map of log2 fold changes in 27 analytes at baseline (corrected by the average levels in patients without CHD). **(B–K)** Differentially expressed analytes at baseline in patients with ACS compared with patients without CHD. Data are presented as medians with interquartile ranges. Differences were tested using Mann–Whitney *U*-test.

A high-sensitivity 27-plex assay was performed for the measurement of the plasma concentrations of IL-6, RANTES, VEGF, GM-CSF, IL-1β, IL-13, PDGF-BB, IL-12p (70), IL-1ra, IL-10, IL-15, IFN-γ, IL-5, MCP-1, IL-2, IL-7, IL-8, eotaxin, IL-4, TNF-α, MIP-1β, IL-9, basic FGF, IL-17, IP-10, MIP-1α, and G-CSF in both groups of patients to assess the inflammatory status of patients with ACS. Surprisingly, chemokines, such as RANTES, eotaxin, IP-10, and MIP-1β (Figures 2B,C,H,K), and pro-inflammatory cytokines, including PDGF-BB, TNF-α, VEGF, IL-9, IL-17, and basic FGF (Figures 2D–J), were higher in patients without CHD compared with patients with ACS. Minor differences in other cytokines/chemokines were noted (Figure 2A), but none of them were statistically significant.

Accumulating evidence has implicated the importance of inflammation disorder in atheromatous plaque formation (20, 21). Therefore, the elevated circulating inflammatory factors in non-CHD patients should be considered a hint of the recruitment of activated macrophages, neutrophils, and T cells in the arterial wall during the premature stage of cardiovascular events. These findings suggested that the patients who came to the hospital because of chest pain but diagnosed as non-CHD might have experienced a heavier inflammatory burden due to anxiety compared with the patients with ACS.

### Single-dose evolocumab during hospitalization evidently attenuated lipid burden in patients with ACS

All patients with ACS in this study presented chest pain at admission and underwent PCI, and their blood samples were then collected at baseline (before evolocumab administration) and 72 h after single-dose evolocumab administration. To assess the rapid effect of single dose evolocumab on lipid metabolism and its potential effect on platelet activation, we measured the lipid profiles and platelet activation markers (Supplemental Figure 2), and the data are summarized in Table 2.

**Table 2:** Lipid profiles and platelet activation markers of patients with ACS at baseline and 72 h post-evolocumab administration.

	**Baseline** **(*n* = 31)**	**Evolocumab** **(*n* = 31)**	***P*** **-value**
TG (mg/dL)	152.4 [113.4, 217.1]	129.4 [108.1, 170.1]	0.0082
TC (mg/dL)	194.1 [159.3, 221.9]	100.9 [71.91, 143.0]	<0.0001
HDL-C (mg/dL)	36.73 [32.86, 46.39]	36.73 [31.31, 44.07]	0.0868
LDL-C (mg/dL)	109.0 [90.85, 144.6]	41.37 [19.33, 69.97]	<0.0001
sdLDL-C (mg/dL)	34.02 [29.38, 52.19]	12.76 [8.119, 17.78]	<0.0001
ApoA1 (g/L)	1.23 [1.12, 1.39]	1.19 [1.05, 1.34]	0.0272
ApoB (g/L)	1.06 [0.92, 1.19]	0.51 [0.31, 0.72]	<0.0001
ApoE (g/L)	4.12 [3.30, 5.17]	2.36 [1.69, 3.18]	<0.0001
Soluble P-selectin (ng/mL)	26.03 [20.04, 33.06]	29.40 [24.60, 37.14]	0.2132
Platelet factor-4 (ng/mL)	1.38 [1.15, 1.65]	1.21 [0.81, 1.66]	0.1270

*Data expressed as median (interquartile range). Differences were tested using Wilcoxon matched-pairs signed rank test*.

*TG, triglyceride; TC, total cholesterol; HDL-C, high-density lipoprotein cholesterol; LDL-C, low-density lipoprotein cholesterol; sdLDL-C, small dense low-density lipoprotein cholesterol; Apo, apolipoprotein*.

**Figure 3:**
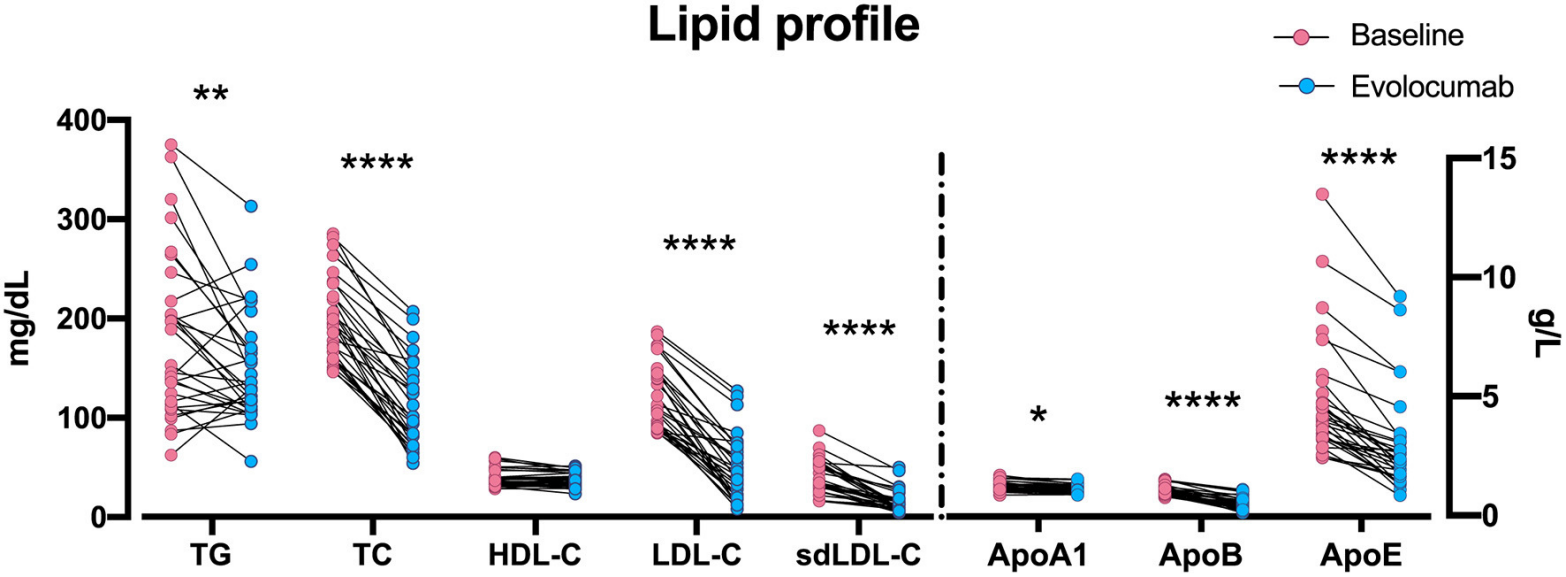
Changes in lipid particles at 72 h post-evolocumab administration. Scattered plot of lipid profile data in Table 2. The dots of TG, TC, HDL-C, LDL-C, and sdLDL-C are aligned to the left Y axis, and the dots of Apo-A1, Apo-B, and Apo-E are aligned to the right Y axis. *P < 0.05, **P < 0.01, ****P < 0.0001. Differences were tested using Wilcoxon matched-pairs signed rank test. TG, triglyceride; TC, total cholesterol; HDL-C, high-density lipoprotein cholesterol; LDL-C, low-density lipoprotein cholesterol; sdLDL-C, small dense low-density lipoprotein cholesterol; Apo, apolipoprotein.

We observed a decrease of plasma PCSK9 in patients with ACS from 337.7 ± 83.3 ng/mL at baseline to 297.9 ± 85.8 ng/mL at 72 h post single-dose evolocumab (Supplemental Figure 1). The median LDL-C level of the patients with ACS was remarkably reduced from 109.0 to 41.4 mg/dL as early as 72 h after single-dose evolocumab, achieving a 60.5% average LDL-C reduction. The efficacy was comparable to previous evolocumab trials, where ~60% LDL-C reduction from baseline was reached on at least week 4 (13, 17, 18, 22, 23). Notably, evolocumab also remarkably diminished other atherogenic lipid particles, with average reductions of 18.1, 43.8, 60.6, and 49.5% in TG, TC, sdLDL-C, and ApoB, respectively. A remarkable decrease in ApoE, minor changes in ApoA1, and no remarkable difference HDL-C were found (Figure 3).

All patients with ACS were prescribed high-intensity statin therapy (rosuvastatin 10 mg/day) without regular evolocumab at discharge and 23 of them were successfully followed up. The median follow-up time was 10.7 months and the median LDL-C levels rebounded from 45.5 mg/dl at discharge to 70 mg/dL. None of these patients experienced MACEs, liver or kidney dysfunction during our follow-up.

### Evolocumab altered inflammatory status in patients with ACS in the early period

Given the rapid and evident effect of evolocumab in lipid burden reduction, and previous hypothesis on the anti-inflammation potential of PCSK9 inhibitors, the levels of circulating inflammatory biomarkers before and after evolocumab treatment in patients with ACS were compared.

**Figure 4:**
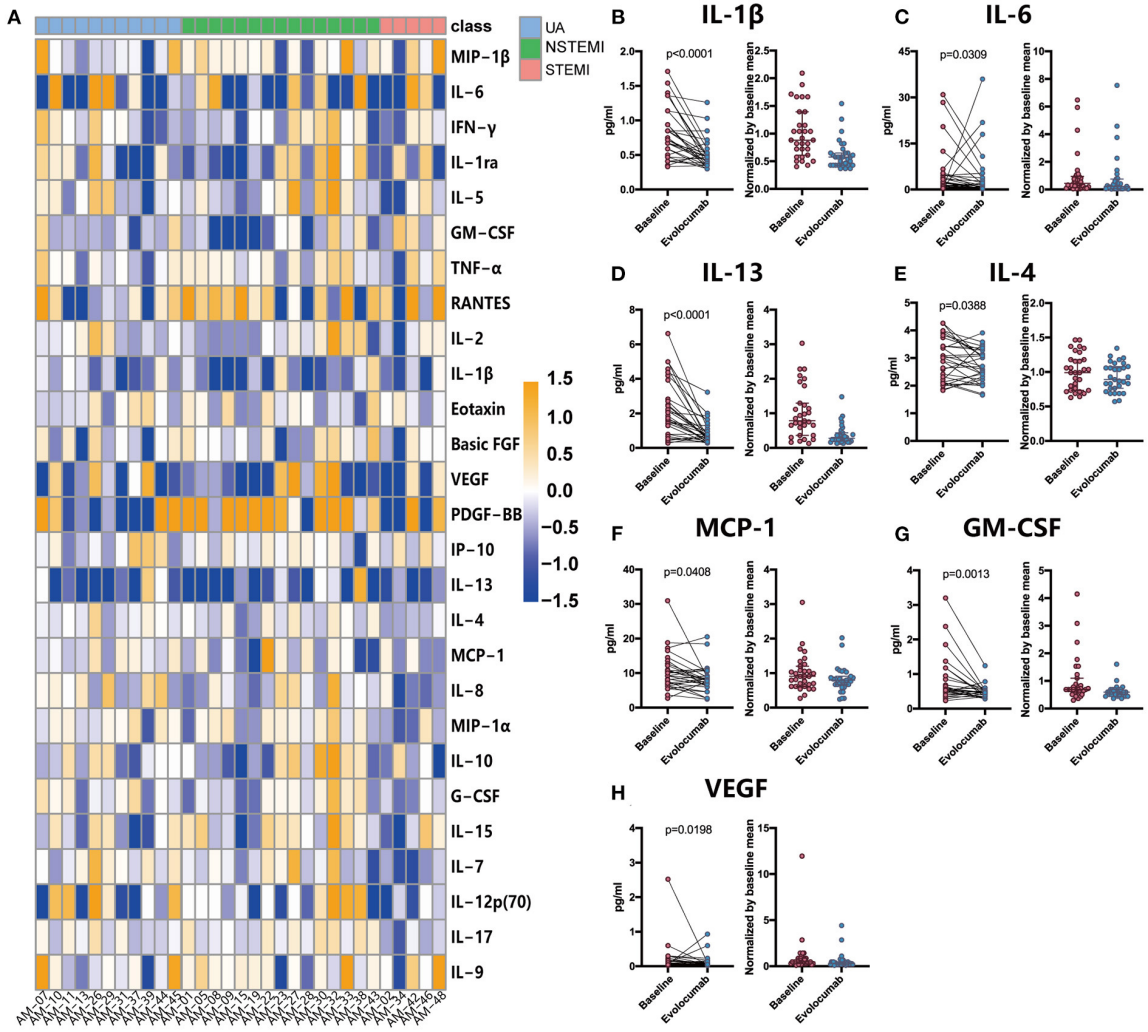
Comparison of plasma cytokine and chemokine levels between baseline and 72 h post-evolocumab administration in patients with ACS. Cytokine and chemokine levels were measured in 31 ACS patients using Luminex assay. **(A)** Heat map of log2 fold changes over the average baseline levels of 27 analytes. **(B–H)** Differentially expressed cytokines and chemokines. Differences were tested using Wilcoxon matched-pairs signed rank test. Data are shown as actual concentrations (left) or medians with interquartile ranges of values normalized by baseline mean (right). UA, unstable angina; NSTEMI, non-ST-elevation myocardial infarction; STEMI, ST-elevation myocardial infarction.

As shown in Figure 4, pro-inflammatory cytokine IL-1β was significantly decreased after evolocumab administration compared with baseline (0.47 [0.35, 0.53] pg/mL vs. 0.72 [0.50, 1.14] pg/mL, *P* < 0.0001), and a similar pattern was observed in IL-6 (1.11 [0.29, 3.63] pg/mL vs. 2.12 [0.68, 4.51] pg/mL, *P* = 0.0039). IL-13 and IL-4, which are considered anti-inflammatory cytokines, were reduced as well after evolocumab treatment (0.61 [0.43, 0.96] pg/mL vs. 1.72 [0.79, 2.83] pg/mL at baseline, *P* < 0.0001 for IL-13; 2.59 [2.22, 3.14] pg/mL vs. 2.88 [2.13, 3.43] pg/mL at baseline, *P* = 0.0388 for IL-4).

MCP-1, a chemotactic molecule for monocytes/macrophages, was slightly downregulated (8.07 [6.79, 9.20] pg/mL vs. 9.20 [6.22, 12.19] pg/mL at baseline). A minor decrease was also noted in VEGF (0.06 [0.04, 0.10] pg/mL vs. 0.10 [0.06, 0.18] pg/mL at baseline, *P* = 0.0013) and GM-CSF (0.46 [0.34, 0.53] pg/mL vs. 0.53 [0.46, 0.85] pg/mL at baseline, *P* = 0.0198). No remarkable changes were observed in the remaining cytokines, including TNF-α and IFN-γ. The differentially expressed cytokines are summarized in Table 3.

**Table 3:** Concentrations and percentage changes of differentially expressed cytokines.

	**Baseline** **(*n* = 31)**	**Evolocumab** **(*n* = 31)**	**% Change from baseline**	***P*** **-value**
**Pro-inflammatory cytokines**				
IL-1β (pg/ml)	0.72 [0.50, 1.14]	0.47 [0.35, 0.53]	−37.7% (−49.2 to −14.6%)	<0.0001
IL-6 (pg/ml)	2.12 [0.68, 4.51]	1.11 [0.29, 3.63]	−60.5% (−76.5 to −15.9%)	0.0309
MCP-1 (pg/ml)	9.20 [6.22, 12.19]	8.07 [6.79, 9.20]	−15.9% (−32.8 to 13.8%)	0.0408
**Anti-inflammatory cytokines**				
IL-13 (pg/ml)	1.72 [0.79, 2.83]	0.61 [0.43, 0.96]	−57.6% (−75.5 to −41.5%)	<0.0001
IL-4 (pg/ml)	2.88 [2.13, 3.43]	2.59 [2.22, 3.14]	−10.1% (−19.6 to −2.2%)	0.0388
**Growth factors**				
GM-CSF (pg/ml)	0.53 [0.46, 0.85]	0.46 [0.34, 0.53]	−26.1% (−30.6 to −13.2%)	0.0013
VEGF (pg/ml)	0.10 [0.06, 0.18]	0.06 [0.04, 0.10]	−40.0% (−60.0 to 0.0%)	0.0198

*Data expressed as median [interquartile range] for cytokine concentrations and as median difference with 95% confidential interval for % change from baseline, respectively. Differences were tested using Wilcoxon matched-pairs signed rank test*.

## Discussion

This study is one of the first studies focusing on immediate lipid-lowering efficacy and systematic inflammatory pattern changes mediated by the in-hospital use of evolocumab in Chinese patients with ACS. In our study, we showed that:

1) The in-hospital use of single-dose evolocumab (140 mg) on top of high-intensity statin resulted in a universal reduction in atherogenic lipids as early as 72 h in patients with recent ACS, and
2) Treatment with evolocumab lowered the circulating levels of pro-inflammatory cytokines, such as IL-1β, IL-6, and MCP-1; anti-inflammatory cytokines, such as IL-13 and IL-4; and growth factors, such as GM-CSF and VEGF.

To date, the lipid management strategy for patients with ACS is stepwise and characterized by the initiation of high-intensity statin, succeeded by the addition of ezetimibe, and the administration of PCSK9 inhibitors only when the LDL-C level remains above the threshold (1, 2). Following this approach, PCSK9 inhibitors would not be considered until several months after an index event. However, the risk of recurrent ischemic events remains the highest during early post-ACS (24) probably because of the continued elevation of LDL-C level and overwhelming inflammation (25). Thus, a rapid lipid-lowering and inflammation-controlling approach is of great importance during acute-phase post-ACS.

The Evolocumab for Early Reduction of LDL Cholesterol Levels in Patients with Acute Coronary Syndromes study showed that the early initiation of evolocumab in patients with ACS exerted a more rapid and powerful LDL-C reduction within 4 weeks and enabled more than 95% patients to reach guideline-recommended LDL-C targets without considerable adverse events (17). Similarly, in the Evolocumab in Acute Coronary Syndrome study, patients with non-ST-elevation myocardial infarction (NSTEMI) who received one dose of evolocumab in the hospital exhibited a substantial decrement in LDL-C level from baseline within 1 day and a remarkable downward trajectory throughout their hospitalization (18). Consistently, in our study, early intervention with single-dose evolocumab (140 mg) remarkably alleviated the atherogenic lipid burden as early as 72 h. Notably, the reduction in LDL-C levels in our study reached 60% at day 3, which indicates that the immediate efficacy of evolocumab in our cohort was equivalent to that (at least 4 weeks after evolocumab administration) reported in other studies (13, 17, 18, 22, 23). This exciting finding may be attributed to the relatively low body mass index of Chinese people. We also observed a rebound in LDL-C levels when patients were treated with high-intensity statin alone during our follow-up, which indicates the single-use of evolocumab in addition to statins provides a rapid and strong but short-lived lipid-lowering effect.

Recent trials investigating anti-inflammatory agents in the secondary prevention of MACE have shed light on the advantages of inhibiting inflammation in patients with ACS (26, 27). In the Canakinumab Anti-Inflammatory Thrombosis Outcome Study, targeting IL-1β benefited patients with previous myocardial infarction (MI) in the view of non-fatal MI, non-fatal stroke, and cardiovascular death (26). Notably, among these patients, those who reached a lower level of IL-6 were even less vulnerable to MACEs (28). The Colchicine Cardiovascular Outcomes Trial revealed the protective role of colchicine for patients with recent MI in the aspect of recurrent MACEs by acting on multiple inflammatory pathways.

Previous studies showed that PCSK9 antibodies have no effect on circulating pro-inflammatory cytokines (29). Here, we observed a decrease in pro-inflammatory cytokines (IL-1β, IL-6, and MCP-1) and anti-inflammatory cytokines (IL-4 and Il-13) 72 h after evolocumab administration. Growth factors, VEGF and GM-CSF, which are considered pro-inflammatory (30, 31), declined as well shortly after evolocumab administration. These findings for the first time proved the impact of PCSK9 monoclonal antibody on systematic inflammatory cytokines for patients with recent ACS. Of interest, evolocumab switched the inflammatory status to a less aggressive stage, characterized by a lower level of pro- and anti-inflammation cytokines, implicating the potential role of PCSK9 antibody in regulating immune cell activation.

This preliminary study is limited by the cohort size and the lack of placebo comparison. Given the fact that all patients in this study were on high-intensity statin throughout their hospitalization, the net effect of evolocumab on LDL-C and other atherogenic lipids during this period was not clear. Besides, statins may be also partially involved in the switch of inflammatory status (32, 33). The clinical translation of our finding was also restricted because of the missing follow-up data after discharge. Further investigation on a larger population with placebo group is needed for a better understanding of the inflammation-regulating role of evolocumab at early-stage post-ACS.

## Conclusion

**Figure 5:**
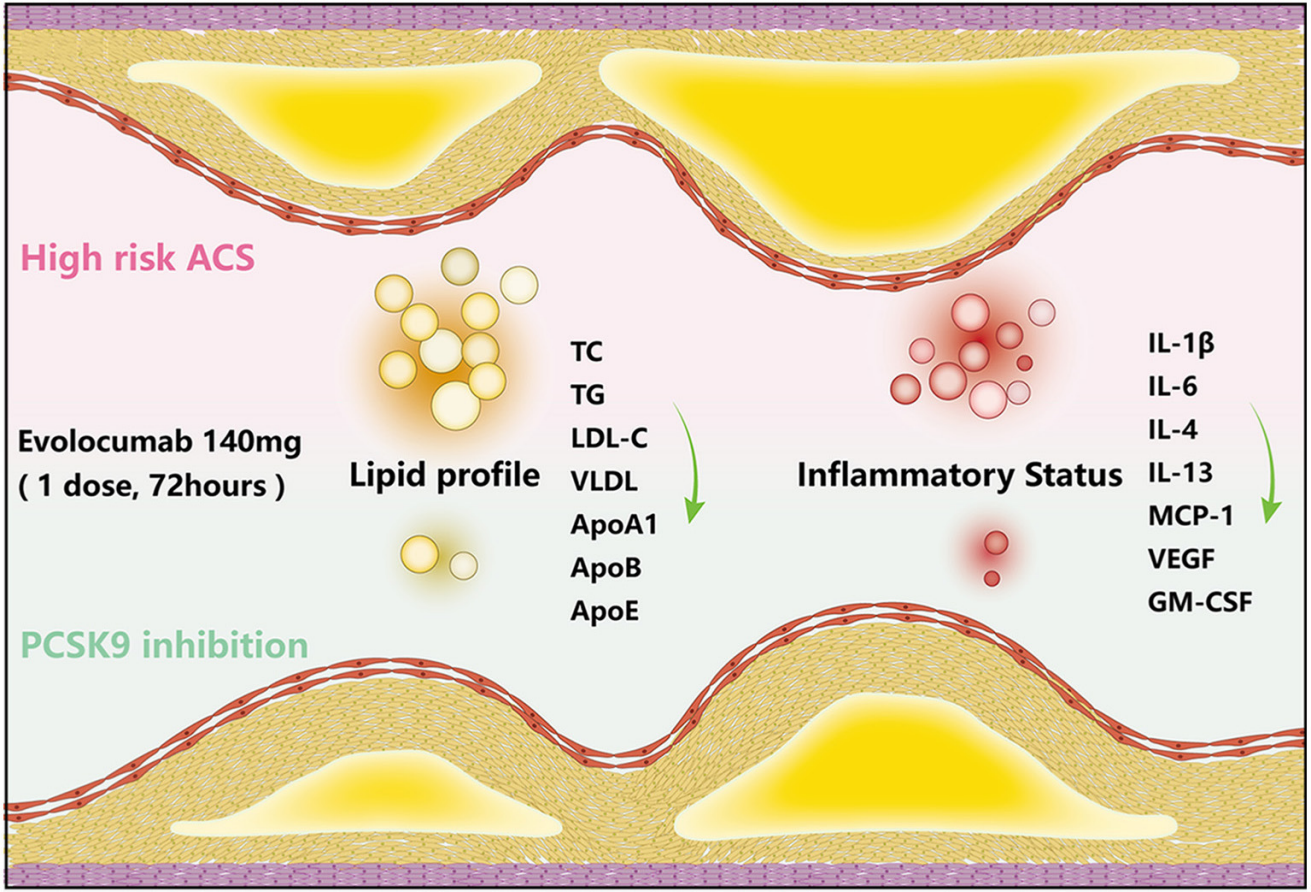
Short-term LDL-C-lowering efficacy of evolocumab in AMONG-US study and schematic diagram. Schematic diagram of the lipid-lowering and inflammation-regulating effects of evolocumab. The levels of TG, TC, LDL-C, sdLDL-C, ApoA1, ApoB, and ApoE, as well as the circulating levels of IL-1β, IL-6, IL-4, IL-13, MCP-1, VEGF, and GM-CSF, decreased at 72 h post-evolocumab administration compared with baseline in patients with ACS.

The prompt use of evolocumab on top of high-intensity statin therapy during early post-ACS enabled patients to achieve rapid lipid burden reduction. Decreased circulating inflammatory cytokines were observed in the patients with ACS treated with evolocumab at early-stage post-index event, indicating a switch to systemic placid inflammatory status (Figure 5).

## Data availability statement

The raw data supporting the conclusions of this article will be made available by the authors, without undue reservation.

## Ethics statement

The studies involving human participants were reviewed and approved by Ethics Committee of Shenzhen People's Hospital. The patients/participants provided their written informed consent to participate in this study.

## Author contributions

TL, SD, XS, LZ, JL, XP, QL, and CX contributed to conception and design of the study. ZO and TL carried out the experiment. ZO wrote the manuscript with support from ZY, TL, and BL. All authors contributed to manuscript revision, read, and approved the submitted version.

## Funding

This research was supported by the National Natural Science Foundation of China (82000058, 82070517, 81873416, and 82070055), Key Research and Development Program of Hunan Province (2020SK2065), Natural Science Foundation of Shenzhen (JCYJ20190807145015194), and Shenzhen People's Hospital Research Cultivation Project (SYJCYJ202014 and SYLCYJ202119).

## Conflict of interest

The authors declare that the research was conducted in the absence of any commercial or financial relationships that could be construed as a potential conflict of interest.

## Publisher's note

All claims expressed in this article are solely those of the authors and do not necessarily represent those of their affiliated organizations, or those of the publisher, the editors and the reviewers. Any product that may be evaluated in this article, or claim that may be made by its manufacturer, is not guaranteed or endorsed by the publisher.
