# Supplementary figures and images for: Evolocumab enables rapid LDL-C reduction and inflammatory modulation during in-hospital stage of acute coronary syndrome: A pilot study on Chinese patients
Source: Front Cardiovasc Med. 2022 Aug 9;9:939791. doi: 10.3389/fcvm.2022.939791 (PMC9397913; doi:10.3389/fcvm.2022.939791)

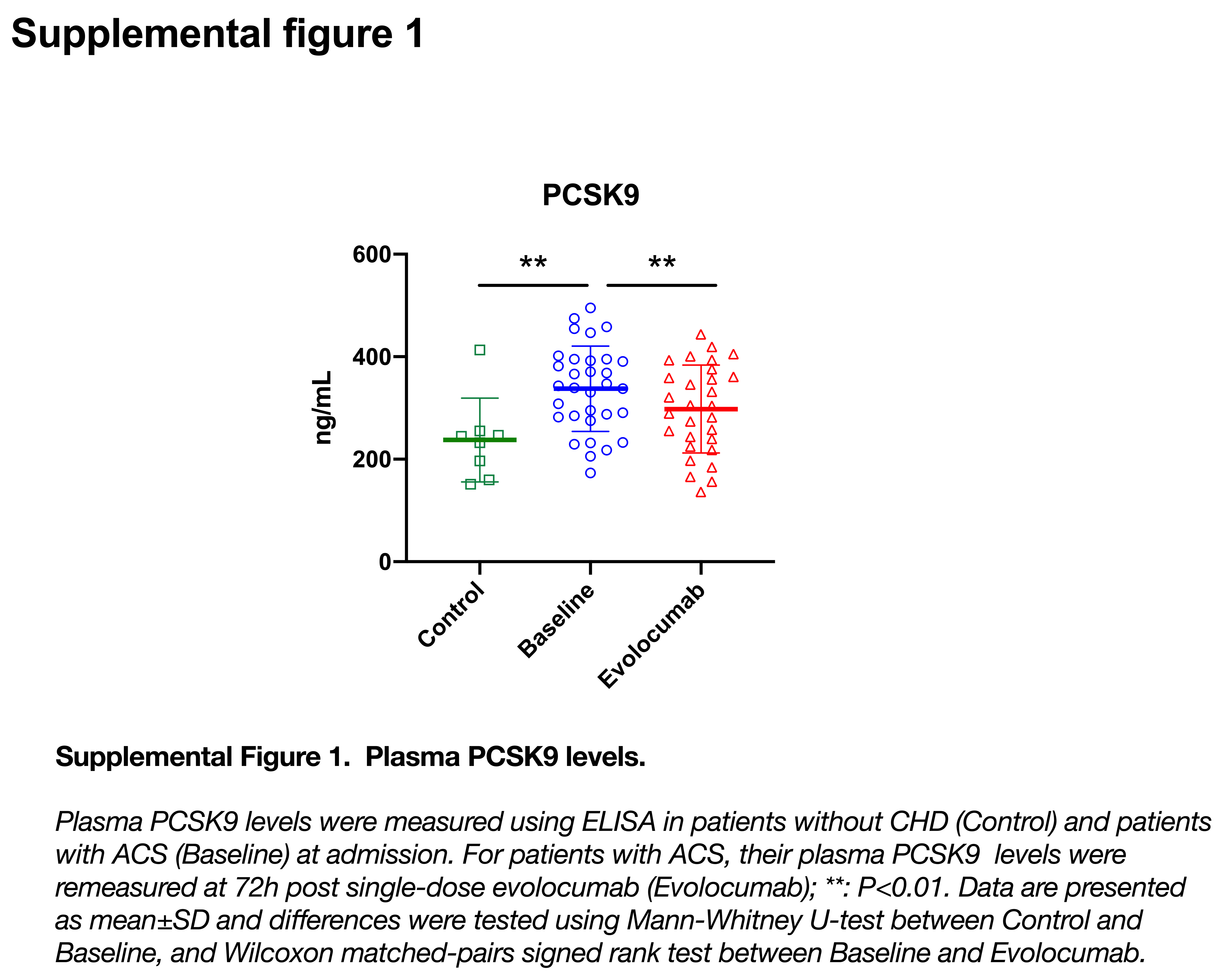

Supplement: Supplementary file 1 [file Image_1.tif]

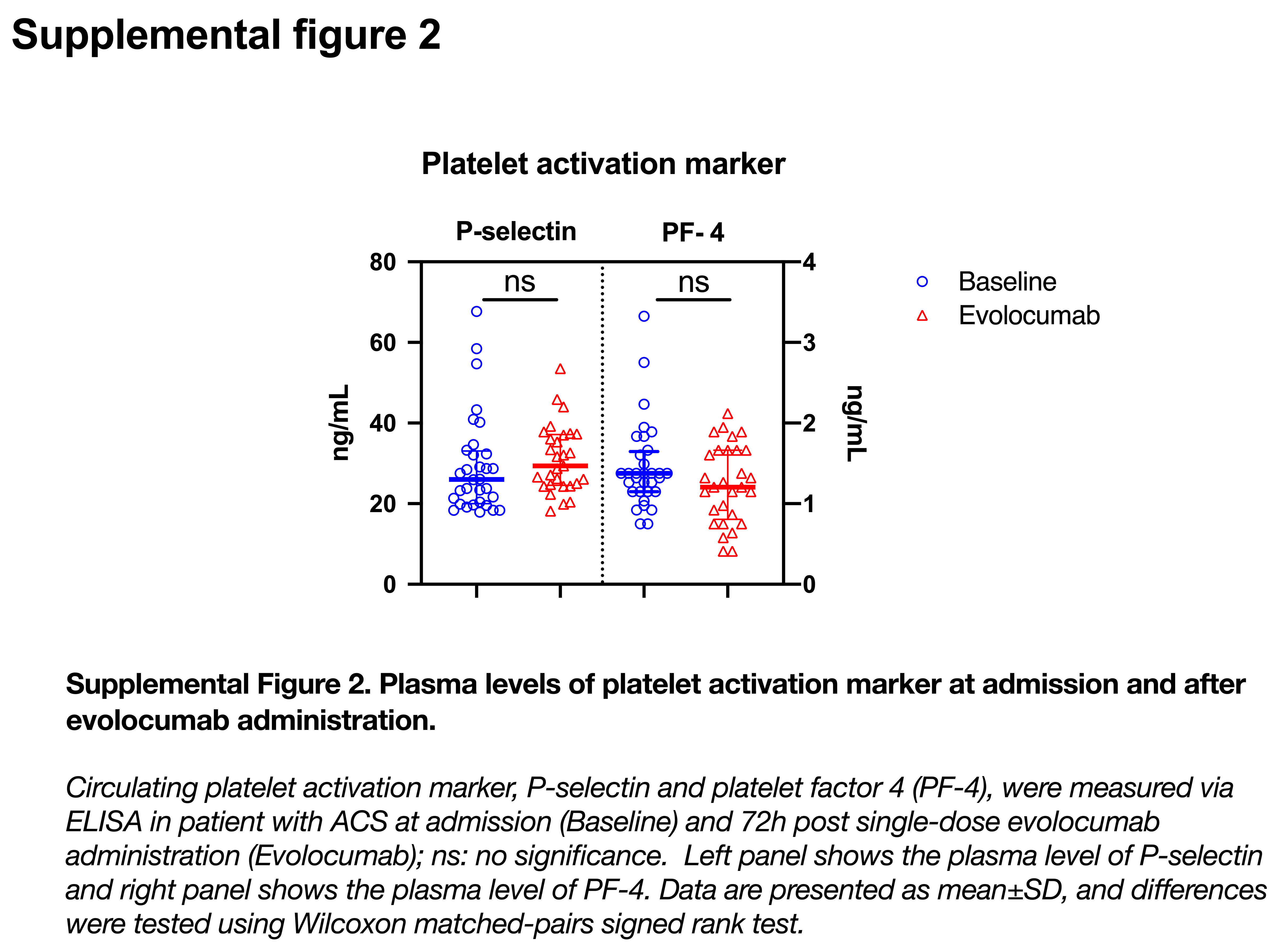

Supplement: Supplementary file 2 [file Image_2.tif]
